# Examining health sector application and utility of program‐based budgeting: County level experiences in Kenya

**DOI:** 10.1002/hpm.3174

**Published:** 2021-05-06

**Authors:** Benjamin Tsofa, Protus Musotsi, Nancy Kagwanja, Dennis Waithaka, Sassy Molyneux, Edwine Barasa, Thomas Maina, Jane Chuma

**Affiliations:** ^1^ KEMRI Wellcome Trust Research Programme KEMRI Centre for Geographic Medicine Research Coast Kenya; ^2^ Department of Public Health School of Human and Health Sciences Pwani University Kenya; ^3^ Centre for Tropical Medicine and Global Health Nuffield Department of Medicine University of Oxford UK; ^4^ The World Bank Group Kenya Country Office Kenya

**Keywords:** health sector planning and budgeting, planning and budgeting, program‐based budgeting

## Abstract

**Introduction:**

In 2012, Kenya enacted a new Public Finance Management Act to guide the public‐sector planning and budgeting process. This new law replaced the previous line item budgeting, with a new program‐based budgeting (PBB) process. This study examined the experience of health sector PBB implementation at the county level in Kenya.

**Methods:**

We carried out a review of the literature documenting the health sector application and utility of PBB in low‐ and middle‐income countries. We then collected empirical data to examine the experience of health sector application of PBB at County Level in Kenya.

**Results:**

In the financial year 2017/18, counties utilised the PBB approach for health sector planning. The PBB approach was perceived by key stakeholders; to have improved the alignment of technical priorities with budgetary allocation, and to have increased transparency, accountability and openness of the process. Its challenges included lack of clear tools and guidelines to support implementation, low capacity at county level, political interference and the organisation of the public sector electronic financial management system around line item budgeting system.

**Conclusion:**

PBB is potentially a useful tool for aligning health sector planning and budgeting and ensuring the Annual Work Plan is more result oriented. However, realisation of this goal would be enhanced by the developing clear tools and guidelines to support its implementation, building capacity for county health sector managers to better understand the PBB application, and reforming the public‐sector budgetary management system to align it with the PBB approach.

## 1 INTRODUCTION

Public sector planning and budgeting can be viewed as a governance and management process for translating policies and long‐term strategic aspirations into day‐to‐day operational activities. Within the health sector it is considered a critical priority setting activity, involving the identification of operational expenditure priorities, allocating resources and determining time frames for activities.^1^, ^2^, ^3^, ^4^, ^5^ The prioritisation and allocation of resources makes health sector planning and budgeting both a technical and political process. The technical elements include setting objectives, goals and targets; while the balancing of different stakeholder interests during the process is a political. The final outputs of the choices made often have economic, political and social ramifications.^1^, ^6^

In the early 2000s, the International Monetary Fund (IMF) and World Bank (WB) introduced and promoted the use of the medium‐term expenditure framework (MTEF) as a framework for guiding public sector planning and budgeting especially in low‐ and middle‐income countries (LMICs). However, even with the wide adoption of these planning and budgeting tools and frameworks in many LMICs, the process of health sector planning and budgeting has often resulted in a mismatch between the technical priority options identified as part of the broader health sector strategic goals, and actual budgetary allocations often because of local political realities and processes.^1^, ^6^

Recent years have seen the introduction and push by global and regional development partners for the adoption of program‐based budgeting (PBB) in LMICs as a tool for facilitating and enhancing the linkage between technical priorities and budgetary resource allocations.^7^, ^8^ PBB involves classifying public sector budgets under programs and subprograms, with each having distinct and specific technical objectives, indicators and targets. Emphasis is placed on the desired output, with information on inputs provided. Several LMICs, including Kenya, Namibia, Mali, Mauritius, Mozambique, Burkina Faso, Zimbabwe and South Africa are already using or are in the process of adopting PBB to guide their public‐sector planning and budgeting.^7^, ^8^, ^9^

In response to a global push by development partners, Kenya as with several other LMICs adopted the MTEF in the early 2000s as a framework for guiding public sector planning and budgeting. In 2005, the MoH introduced the Annual Operational Plans as a way of adapting the MTEF into the health sector planning and budgeting process and assisting the execution of the national health strategic plan.^5^, ^10^ However, even with these reforms, health sector planning and budgeting in the country continued to face significant misalignment between technical priorities and budgetary resource allocation.^2^

Kenya has had a long postindependence history of a centralised government system. However, a clamour for reforms in public sector governance, which peaked in the late 1990s and early 2000s, led to a new constitution being adopted in 2010. The country's 2010 constitution introduced a new devolved system of government with 47 semiautonomous counties.^11^

With devolution, health service delivery functions were allocated to county government while national government retained policy and strategic planning function; as well as national referral services. The county government level has thus become the focal arena for health sector priority setting, planning and budgeting.^12^

In 2012, a Public Finance Management Act (PFMA) was enacted to guide resource allocation, planning and budgeting process; and the overall management of public finance at both the county and national government level. The PFMA 2012 required that both the national and county government institutions shift to PBB for their Annual Work Plans (AWPs) and budgeting. The national government was expected to adopt PBB in the Financial Year (FY) 2013/2014 while the county governments were required to implement it from FY 2014/2015.^13^ With this reform requirement, the national treasury developed its first PBB budget in the financial year 2013/2014. At that time, very few stakeholders understood the process of preparing and reading the budget in this format. As a result, the budget was seen to have errors with some program objectives lacking clarity and useful information missing. Similar inconsistencies were also noticed in the first MoH PBB.^14^, ^15^ Owing to general capacity challenges at county level, and lack of proper guidance from the national level, the implementation of PBB in the health sector at county level only begun in the 2016/17 FY.^14^, ^15^

With the PBB implementation being within its early years of roll out in the health sector and with limited information on its utilisation experience in LMICs available, our study sought to examine the early experiences of county level health sector application of PBB as a planning and budgeting tool and process; and to understand the extent and potential it has had, if any to address the health sector historical challenges of mismatch between planning and budgeting. To the best of our knowledge, this is the first study that has examined the health sector utility of PBB as a public‐sector planning and budgeting approach in LMICs. We undertook this study embedded within the health sector planning and budgeting process and in close collaboration with key stakeholders both at national level. In the process, our study also served as part of an initial process for developing national standardised tools and guidelines for guiding county level health sector PBB in the country.

## 2 STUDY METHODS

This was a multiphase qualitative explorative explanatory study. We undertook data collection in three distinct phases.

### 2.1 Data collection

Phase 1 involved undertaking a scoping review of both published and grey literature to examine any documented experiences of health sector experiences for the application of PBB as a planning and budgeting approach in LMICs. To do this we searched for literature in PUBMED, Science Direct and Google Scholar using the following key words; “Health sector” OR “healthcare” AND “Prog* Based Budget*” OR “Plan* and Budget*” AND “Low and Middle Income Countr*” OR “Developing Countr*” OR “Sub‐Saharan Africa”. We also searched for grey literature from websites of organisations with interest in healthcare planning, budgeting and financing including those of the IMF, WB, World Health Organization (WHO), Collaborative African Budget Reform Initiative and International Budget Partnership. We conducted this search in December 2017 and updated it in March 2018.

For a paper to be included, it had to satisfy the following inclusion criteria; had to be reporting on health sector experience on the application of PBB, be based on LMICs according to the WB classification, be published in English language, and be published (either peer reviewed publication or grey literature) between the year 1990 and 2018. We chose this period because it was during this time that most LMICs started shifting their budgeting formats to PBB.

Preliminary analysis from this phase informed data collection tools development for Phases 2 and 3.

Phase 2 involved conducting a detailed case study in one county as part of an ongoing health system governance long‐term collaborative project we have called a ‘*learning site’*.^16^, ^17^ In this county B. T., P. M., D. W. and N. K. conducted nonparticipant observation of the FY 2018/19 health sector planning and budgeting process, and in‐depth interviews with six purposively selected actors involved in the county PBB process that year. These actors included three County Health Management Team (CHMT) members, one County Treasury official, and two officials of nongovernmental organisations (NGOs) involved in the county planning and budgeting process. We had intended to also interview members of the county assembly (CA) health committee but could not manage after various times of trying to fix appointment and failing. We recorded the key informant interviews using a tape recorder and took summary notes during the interviews which we recorded in field notebooks. B. T., P. M., N. K. and J. C. also reviewed all documents related to the County health sector planning and budgeting process, including the PFMA of 2012, County Government Act 2012, County treasury budgeting circulars, the County Department of Health of Health (CDoH) FY 2017/2018 and FY 2018/19 budgets and AWPs. In addition, and as part of a routine learning site practice, B. T., P. M., D. W., N. K., E. B. and S. M. conducted regular reflective practice sessions among researchers and with selected county level health sector managers.

Phase 3 involved carrying out key informant interviews (conducted by B. T., T. M. and J. C.) with seven select national level stakeholders involved in guiding and supporting the implementation of county level health sector PBB. These included officials of the MoH, officials from the Council of Governors (CoG) secretariat representatives of development partners including the WB and WHO country offices and officials of the Development Partners for Health Kenya. Interviews lasted for between 30 min and 1 h, during which we took detailed notes. We subsequently (in consultation with national level stakeholders) purposefully selected an additional five counties where B. T., T. M. and J. C. proceeded to conduct one focussed group discussion (FGDs) with each respective CHMT and other county level stakeholders, who were at the time participating in the development of the 2018/19 FY AWP. Finally, we conducted a 2‐day ‘validation’ meeting which brought together participants from the MoH, the CoG secretariat, national level health sector development partners and three CHMT representatives from each of the 47 counties.

### 2.2 Data analysis

We read through the field notes and listened to the interview recordings after every interview to familiarise ourselves with the data. We then transcribed all recording verbatim, and typed all filed notes using Microsoft Word. We later imported the data into NVIVO version 12 software for coding and thereafter analysed it using a thematic framework approach.^18^ We developed a coding tree, with the initial broad themes and codes based on the policy triangle framework.^19^ The codes and themes were continuously refined throughout the analysis period.

### 2.3 Ethical considerations

We obtained ethical approval for the study from the Kenya Medical Research Institute (KEMRI) Scientific and Ethical Review Unit (SERU) (KEMRI/SERU/CGMR‐C/099/3539), as part of a broader study for examining and strengthening county level health systems in Kenya. We obtained written informed consent from all individual key informant interviewees, and verbal consent for all those participating in FGDs.

We anonymised the data we collected by assigning codes to respondents and avoided mentioning of specific individuals and counties by name. We stored the collected data on a password‐protected computer.

## 3 STUDY FINDINGS

In this section, we begin by presenting a summary of the literature review findings. We later progress to present our empirical findings focussing on the county level health sector PBB process and content in theory, then progress to present findings on the process in practice, followed by a description of the roles of actors in theory and practice. We finally conclude the result section by highlighting some of the opportunities created by the PBB processes for health sector planning and budgeting; and an overview of some challenges facing its implementation.

## 4 HEALTH SECTOR APPLICATION OF PBB IN LMICs

Our literature search generated 894 records in total, of which 57 were duplicates and were removed. The remaining articles were screened by title, which led to 761 articles being removed. Forty‐four articles were removed by abstract screening. Two articles could not be accessed online while the remaining 30 articles were downloaded and screening by reading the full articles, out of which 27 were removed hence only three articles met the inclusion criteria and were used for our review.

All the three articles were grey literature reports from Africa, focussing on national level health sector experiences with PBB. None reported on subnational level experiences particularly in decentralised settings. The reports noted that early experiences of the health sector application of PBB introduced an improvement in the budget presentation with the budget having technical information, objectives and expected results, indicators of performance and targets to be achieved. These features were absent in the formerly used Line‐Item Budget format.^7^, ^9^, ^20^

## 5 THE COUNTY LEVEL HEALTH SECTOR PBB PROCESS AND CONTENT IN THEORY

From the Kenyan documents we reviewed, County governments were expected to adopt PBB as a planning and budgeting process in the FY 2014/2015. From the guidelines, public sector budgets should be organised around programs with each program having clear priorities, activities (services or goods offered within it), measurable indicators and budgetary allocation. Each planning unit/department should have a minimum of three and maximum of five programs. Respective planning units are required to carry our quarterly reviews of the budget and at the end of the FY to inform the subsequent budget. The generic guidelines however did not provide clear guidance on what should constitute a program or subprogram.^21^

Within the county, at the beginning of each FY, the PBB process requires the CEC member for finance to issue a circular to all county government departments specifying the schedule of the PBB process including key dates and deadlines, policy areas to be considered in the budgeting process, the process of public participation and the format in which budget documents should be submitted. The CEC finance also has a responsibility for ensuring that the budgeting process is conducted in the required manner and the people involved in the process have adequate time to meet the necessary legal requirements. Public participation in the planning and budgeting process is a legal requirement, as is ensuring that the approved budget is accessible to and easily understood by the public.^21^

## 6 THE COUNTY LEVEL HEALTH SECTOR PBB PROCESS IN PRACTICE, AND ITS INFLUENCES

Several counties reported that within their counties, the CEC finance issued a circular to all county departments as is required by law, with broad guidelines on the process, timelines and generic departmental formats for the budget. The circular required departments to focus their budgets on the priorities set out in the respective county government MTEFs, County Integrated Development Plans, and the Kenya Vision 2030 national long‐term development agenda. Departments were also required to carry out detailed reviews of the implementation of the previous year workplans and budgetary allocations before embarking on the preparation of the new one, and to present their budget in PBB format, with clear program indicators focussing on outputs and outcomes that are specific, realistic, achievable and time bound. There were instructions that activities with unclear outputs, indicators and targets would not be considered for funding.

Across counties there was variation in the number of programs identified and planned for within the department of health for the FY 2017/18. These ranged from 2 to 5, with each of the six counties having differences in the program numbers and names. In most counties, it was reported that the CDoH 2017/18 AWPs were developed after the budgetary process had been concluded and that county budgets were approved by respective CAs. In the *learning site* county for example a review of the previous year's (FY 2016/17) budget was delayed, and was finally done at the end of the financial year when the FY 2017/18 budgeting process had been concluded. The CDoH delayed in the preparation of their AWPs and the treasury went ahead and allocated funds to CDoH programs without their (CDoH) participation. This CDoH AWP hence it did not inform budget allocation. A review of the FY 2017/18 AWP noted that it was not complete and did not have any narrative information about the technical priorities identified.

For the FY 2018/19 planning and budgeting cycle, the MoH together with the CoG provided some guidance to the health sector planning and budgeting process, recommending three programs (preventive and promotive services, curative health services and general administration) for uniformity across counties. A harmonised draft CDoH planning and budgeting template was also developed to guide the process in counties. There were varied views across the counties about the idea of having standardised county health sector programs in the planning templates. Some felt that the standardised health sector programs would provide more clarity and uniformity in guiding health sector resource allocation, while others were concerned that having limited and standardised programs for the health sector restricts their ability to focus resources on their county specific priority areas.

*“…. [national] treasury has been very rigid … in terms of the programs. Initially, they were of the position that because we are one country or one government, with 48 sub‐governments, then we need to be like speaking the same language, so, they have like been suggesting that these programs are prescribed, they are already pre‐determined. So, you have to fix or fit within those programs….”* C01 KII 03.

*“……say for example you want to introduce a new programme, you have no room, you have to find a place in that broad aspect…”* C01 KII 01.

The process for the FY 2018/19 was started early in most counties thus, appropriately aligning the planning and budgeting activities. In the learning site county, for example, in the FY 2018/2019, the CDoH with the help of local NGO undertook a review of the early review of the 2016/17 AWP and identified their technical priorities in time to inform the 2018/2019 budget allocation.

*“…. currently, as even as we are discussing now, we have already done our work plan, we have already done our financial year 2018/19 work plan,”* C01 K11 02.

On the process of budget ceiling allocations by county treasuries for both the FY 2017/18 and FY 2018/19, it was reported across counties that the department level (including CDoH) priorities within the county were largely guided by their previous year's expenditures on personnel emolument, resource utilisation capacity of departments, and revenue raising ability of each individual department. Other considerations were the relative *political priority* of the individual department/sector in the county according to the treasury, the governor's pledges to the electorate during the campaign period and the Annual Development Plan.

*“…..there is another component of resource, revenue raising, how much are you contributing to the counties revenue, so that when you raise some, we can give you more……so that we encourage departments to come up with revenue raising mechanisms, and then we give them money…”* C00 FGD.

Although better improved guidance and panning templates were provided for the FY 2018/19, one of the counties we visited reported having added one more program (maternal and child health) in their CDoH budget as they considered it a local priority. The CDOH's AWP and PBB for the FY 2018/2019 in that county was felt to have been more elaborate and good when compared to other departments.

*“We interrogated the programs and we settled for a fourth one. ….. nationally, they were taking like three, the first three, but us as a county we felt, this, the reproductive, maternal and child health. We thought it was a big thing, let's have it, let have programme for it,”* C02 FGD.

*“….By the way the programs for the department of health are so elaborate but what we normally like ask them is, they have programs, sub programs, sub sub‐programs, they can go to the smallest unit of an activity or an output you can ever imagine.”* C002 KII 003.

Several counties also reported a better bottom‐up and inclusive AWP process within the CDoH for the FY 2018/19; with better involvement of primary health facilities and the general public in the process.

## 7 ACTORS AND THEIR ROLES IN THE PBB, IN THEORY AND PRACTICE

Across counties, it was reported that the departments of health's AWP processes were coordinated by the CEC health through the County Director of Health while budgeting was spearheaded by the Chief Officer Health. Although these two processes should be linked, their coordination by different CDoH actors undermined this linkage during the FY 2017/18. However, with the introduction of more harmonised PBB tools and guides in the FY 2018/19, all the six counties reported better harmonisation and alignment of planning and budgeting processes.

Across the six counties it was reported that the CHMTs, Subcounty Health Management Teams (SCHMTs), representatives from the county and subcounty hospitals and some primary health facilities were involved in the AWP process in the FY 2018/19.

Local level political players also played a major role in the resource allocation process, with health committees in CAs having a significant influence on the CDoH planning and budgeting process and general resource allocation.

*“……, the development budget has a lot of input from the MCAs [* Members of County Assembly]*. Because this guy wants a dispensary here and not on the other side. And remember, in this political dispensation, MCAs get changed, they get elected, maybe a new one comes, the other one built a dispensary here, and this other one also wants a dispensary.”* C003 FGD.

The legal frameworks require active public participation in the budgeting process through the sector budgeting forum. However, across counties it was felt that public inputs beyond MCAs were not incorporated in FY 2018/19 because of the technical nature of health sector planning. In one county FGD, it was reported, for example, that the public gave wishlists without considering objectivity.

The other key player in the CDoH PBB process in theory as highlighted in the guidelines, should be the County Economic Budgeting Forum; which is a constitutional body gazetted by a respective governor made up of experts from different professional and advocacy groups. In theory, this forum should play a role in assisting the county make its budget. However, we did not get data of its existence and activities in practice in any of the six counties. In addition, local NGOs and other health stakeholders variedly assisted the CDoHs in preparing their AWPs and budgets.

## 8 THE PBB OPPORTUNITIES, OUTCOMES AND CHALLENGES

Across counties there was a strong feeling that the CDoH AWP for the FY 2018/19 was prepared on time, and that the budget was better aligned to the technical priorities identified during the planning process than in FY 2017/18. In one of the counties however, the FGD participants felt that, even with the improvement in alignment, the newly prioritised activities in the AWP were unlikely to be funded:

*“…, we have a lot of historical garbage* [debt]*, so, we have focused more on clearing the historical garbage* [debts], *or rather on the previous year's garbage* [debts]*….. in fact we want to bring on board another, another approach in budgeting, what we want to call zero based budgeting, [where the goal is to] clear most of the* debts that we have had*,”* C001 FDG.

Most stakeholders across counties viewed PBB as a useful planning framework for guiding resource allocation, decision‐making and aligning the CDoH AWP with budget allocation. It was felt that PBB made it easier to track expenditure and carry out monitoring and evaluation and improved implementation of prioritised activities. In addition, PBB process was felt to have increased openness of the budget to the public, and increased transparency and accountability. It was also said to have increased consultation during the budget making process and to have been a useful tool for mobilizing for resources from partners.

“*…it has promoted transparency…. Just a few weeks [ago], I talked to somebody from here {county name}. They had gone for a public participation and transparency [and were asked]: ‘Before you had promised to do ABCD, but now, you have only done a bit of what you promised to do’. “Last time you were allocated seven million, how come this time round you still want to allocate more resources for that program yet we don't see anything worth the seven million?’ …I think going with this approach will make the department be more accountable first to themselves as the department and to the people”*. C03 FGD.

However, across counties, early implementation of PBB also faced challenges. First, the transition from line item to PBB was not fully optimised. In all the study counties, a key concern reported was that the national Integrated Finance Management Systems (IFMIS) managed by national treasury and which is used by all public entities including county governments, was still organised in a Line‐Item Based manner. Secondly, most CDoH officials involved in the PBB process did not have adequate knowledge about it. In addition, the PBB process resulted in a new role for the general public and political leaders in financial planning and budgeting; which health managers were not used to previously.

In all the six counties, another key concern reported that the finance management payment functions were centralised at the county treasury rendering the CDoH unable to play a role on prioritisation of expenses and servicing of debts and on‐going expenditure leading to delays in budget execution. The delay and nonpayment for services offered to the CDoH by respective county treasuries was reported to have been affecting of the CDoH AWPs implementation.

*“…we have not decentralized the treasury, everything goes there…. So, the guy who is paying. he has the muscle this guy. As we know, this is priority, Daktari* [Doctor] *here knows this is priority, we have planned for blood, we have planned this, this is critical. But the guy looks at the voucher and says, ok, I will pay this tomorrow. You see, who has failed? Me I have done my part, but I am waiting, so I become a beggar at the end of the day….”* C01 KII 02.

## 9 DISCUSSION

PBB is still new as a public‐sector planning and budgeting framework in Kenya, having been adopted in the country a few years ago. It was adopted in the Kenyan health sector at a point when knowledge and understanding of it application by key sector actors was very low, and during a major political transition from a centralised to a devolved government system. The early years of its application in the Kenya health sector at county level saw counties adopt different numbers and definitions of health sector programs. In addition, other challenges that affected its implementation at county level in the early days was the lack of operational tools and guidelines to guide county health sector managers on its implementation; and the nonconformity of the treasury IFMIS system that's is still organised around line‐item budgeting framework. However, the early days of health sector implementation of PBB saw stakeholders reporting better public participation and transparency in health sector planning and budgeting, and better alignment of health sector technical priorities and budgetary resource allocation.

The health sector knowledge and capacity challenge in implementing PBB has also been reported in other settings in Africa.^22^ This challenge may in part be linked to the limited studies and published data available on the application of this planning and budgeting framework. There is scanty literature or well modelled PBB from which the LMICs adopting it can learn.^23^

PBB was introduced as an opportunity to solve the problem of historical misalignment between planning and budgeting in the public sector, including the health sector, when it became clear that earlier MTEF and AWPs frameworks had failed to strengthen alignment.^2^ The PBB planning and budgeting framework was designed to link planning and budgeting and serve as an integrating framework for the two processes that had operated separately for many years. We found significant achievements in this broad goal of aligning health sector planning and budgeting with the adoption of PBB. PBB was also reported by county level stakeholders to have improved the ability of the health sector to track expenditure and carry out monitoring and evaluation; though we could not obtain any tangible data to confirm this. There was also reported increased openness of the budget to the public, and increased transparency and accountability.

The county level health sector implementation for PBB was however facing some challenges. The transition from line item to PBB was not fully optimised with the treasury IFMIS finance management system still being reported to be Line‐Item based. Most CDoH officials involved in the process did not have the capacity in the format and its legal framework. PBB resulted in a new players and roles in financial planning and budgeting in the health sector which county health managers were not used to.

Across the various counties involved in the study, the CDoH have made some improvements over time to their planning process following the adoption of PBB; with the FY 2018/2019 cycle being reported to having been done in time to inform budget allocation as opposed to after budget approval, which has been observed in the Kenya health sector in earlier studies.^4^, ^12^, ^24^ This reported improvement was attributed by county level stakeholders to be due to the application of the PBB process; and was considered a significant step towards addressing the health sector historical challenge of planning and budgeting misalignment. However, our study suggests that this achievement is undermined by a centralised county treasury system where county treasuries prioritise all county government expenditures and payments without involving the respective departments.

Although the process of AWP preparation and PBB should be bottom up as envisaged in law, this was not the case across the study counties. In all of them, the process was majorly driven as top down by respective CHMTs. This finding resonates with those of other studies on health planning and budgeting done in Kenya.^2^, ^25^ However, the introduction of MCAs and their roles as part of the broader devolved government created opportunities for inclusion of public voices in the health sector planning and budgeting process.

Although the CDoH PBB process was not fully inclusive across counties with primary health facilities not involved; the general number of stakeholders involved in the health planning and budgeting process with PBB was reported to have increased, with more public and local politicians involved; compared to what used to happen previously. This was in agreement with what was found in a study on county level health sector priority setting where the stakeholders involved in planning and budgeting were reported to have increased since the use of PBB was initiated.^4^ However, increased stakeholders' engagement in health sector planning and budgeting has its challenges. Gray and Steele argued that with many players involved in the decision‐making, they can easily lose focus of the main objectives and priorities.^26^

From our study findings, not all systems involved in public sector finance management transitioned to PBB. The treasury IFMIS system for budgetary execution was reported to be still operating on a line‐item budget system. This similar challenge was also noted in other African countries that had introduced PBB^22^; and is in agreement with observations made by Kim in Korea who noted challenges in national treasuries fully restructuring existing systems and structures to conform to PBB requirements.^23^

Failure by the treasury to decentralise the various financial management functions to the respective departments across counties was a serious hindrance to PBB implementation. The PBB framework requires the various program managers to be accountable for performance in their respective programs. However, with the lack of control over the expenditures of allocated budgets for the various health sector programs, the program managers lacked ability to fully direct the program implementation and hence be fully accountable for full program implementation.

## 10 CONCLUSION AND RECOMMENDATIONS

This study contributes to the scarce empirical literature on PBB experience in the health sector. We conclude that the early days of PBB application in the Kenyan health sector at county level is showing very useful utility and potential of this framework in addressing the historical challenges of lack of linkage between technical planning and budgeting. In addition, PBB has a potential of increasing transparency and openness in public health sector resource allocation and budgeting process; and in making the budgeting process results‐oriented. These are important potential gains in strengthening of health sector resource allocation and priority setting which is going to be a critical enabler in the country's journey towards Universal Health Coverage. However, the potential and utility of PBB at the county level in Kenya is yet to be fully realised due to various systemic implementation level challenges. To address these, we would like to make the following recommendations.

From the lessons learnt in the Kenyan experiences, it would be important for LMICs adopting or implementing PPB to do develop clear and explicit guidelines for the implementation; and to update their financial management systems to align them with the PBB format.

In Kenya specifically, there is need for the national government (MoH and national treasury), to urgently update the IFMIS system to align it with the PBB format. There is also a need for updating the sector level PBB application guidelines to provide more elaborated guide to the definition and establishment of programs and subprograms at sector level. In addition, it could be worth providing some more flexibility in the elaboration and number of programs for different sectors to allow for county managers better align the PBB tools to local county needs. Continuous capacity building for county health managers and other stakeholders on the PBB process is also needed. At county level, there is need to open, and proactively involve members of the general public and other stakeholders to take part in the PBB process.

For the research community, though in its early days, PBB is showing early signs of strengthening health sector planning and budgeting processes. There is however need for more studies to document and evaluate the utility of this budgeting framework for the health sector in different settings across LMICs.

## Data Availability

Data that may be made available include data included in the manuscript in form of quotes; summaries of the main themes; and anonymized data transcripts of participant interviews and group discussion, in keeping with the KEMRI‐Wellcome Trust Research Programme's Data Governance Policy. Requests can be facilitated by contacting the KWTRP's Data Governance Committee on email at: dgc@kemri-wellcome.org.

## References

[1] Kirirgia J , Sambo L , Agu V , Lambo E . How to develop an operational plan for health. East Afr Med J. 2001;78(3):14‐19. 12002062 10.4314/eamj.v78i3.9071

[2] Tsofa B , Molyneux S , Goodman C . Health sector operational planning and budgeting processes in Kenya‐"never the twain shall meet". Int J Health Plann Manage. 2016;31(3):260‐276. 25783862 10.1002/hpm.2286 PMC4988384

[3] Tsofa B , Molyneux S , Gilson L , Goodman G . How does decentralization affect health sector planning and financial management? A case study of early effects of devolution in Kilifi County, Kenya. Int J Equity Health. 2017;16:151. 28911325 10.1186/s12939-017-0649-0 PMC5599897

[4] Waithaka D , Tsofa B , Kabia E , Barasa B . Describing and evaluating healthcare priority setting practices at the county level in Kenya. Int J Health Plan Manage. 2018;33(3):e733‐e750. 10.1002/hpm.2527 PMC6120533 29658138

[5] Tsofa B , Molyneux S , Goodman C . Health sector operational planning and budgeting processes in Kenya‐"never the twain shall meet. Int J Health Plan Manage. 2016;31(3):260‐276. 10.1002/hpm.2286 PMC4988384 25783862

[6] Green A , Mirzoev T . Planning, for Public Health Policy. 2008.

[7] Collaborative African Budget Reform Initiative (CABRI) . Performance and Programme‐Based Budgeting in Africa: A Status Report. 2013.

[8] Lakin J , Torbert S , Hasan S . Program Budget Structure in the Health Sector : A Review of Program‐Based Budgeting Practices in Low‐ and Middle‐Income Countries International Budgets Partnerships; 2018.

[9] Magero V , Lakin J . Improving Program‐Based Budgeting in Kenya. 2015.

[10] Oyaya CO , Rifkin SB . Health sector reforms in Kenya: an examination of district level planning. Health Policy. 2003;64(1):113‐127. 12644333 10.1016/s0168-8510(02)00164-1

[11] Government of Kenya . Constitution of Kenya. Revised Edition. Nairobi, Kenya: The National Council for Law Reporting; 2010.

[12] Tsofa B . Examining the Effects of Political Decentralisation in Kenya on Health Sector Planning and Budgeting: A Case Study of Kilifi County. London School of Hygiene & Tropical Medicine; 2017.

[13] Government of Kenya . The_Public_Finance_Management_Act_2012. National Council for Law Reporting; 2012.

[14] Government of Kenya . Programme Based Budget For The National Government. In: Treasury N , ed. Nairobi, Kenya: National Treasury; 2013.

[15] Lakin J , Magero V . Improving Program‐Based Budgeting in Kenya. International Budget Partnership Organisation. 2015.

[16] Nyikuri M , Tsofa B , Barasa E , Okoth P , Molyneux S . Crises and resilience at the frontline—public health facility managers under devolution in a sub‐county on the Kenyan Coast. PLoS One. 2015;10(12):e0144768. 26696096 10.1371/journal.pone.0144768 PMC4687913

[17] Resilient, Responsive Health Systems (RESYST) . What is a Health System Learning Site?. http://resyst.lshtm.ac.uk/2016http://resyst.lshtm.ac.uk/resources/podcast‐what‐health‐system‐learning‐site. Accessed March 20, 2017.

[18] Pope C , Zielbland S , Mays N . Qualitative research in health care: analysing qualitative data. BMJ. 2007;320:114‐116. 10.1136/bmj.320.7227.114 PMC1117368 10625273

[19] Walt G , Gilson L . Reforming the health sector in developing countries: the central role of policy analysis. Health Policy Plan. 1994;9(4):353‐370. 10139469 10.1093/heapol/9.4.353

[20] Lakin J , Magero V . Understanding Program‐Based Budgeting: Toward Improved Budget Transparency in Kenya. International Budget Partnership; 2014.

[21] Government of Kenya . The Public Finance Management Act. National Council for Law Reporting; 2012.

[22] CABRI . Performance and Programme‐Based Budgeting in Africa: A Status Report. Pretoria: CABRI; 2013.

[23] Kim JM . From Line‐item to Program Budgeting: Global lessons and the Korean Case. Korea Institute of Public Finance; 2007.

[24] Barasa EW , Cleary S , Molyneux S , English M . Setting healthcare priorities: a description and evaluation of the budgeting and planning process in county hospitals in Kenya. Health Policy Plan. 2017;32(3):329‐337. 27679522 10.1093/heapol/czw132 PMC5362066

[25] O'Meara WP , Tsofa B , Molyneux S , Goodman C , McKenzie FE . Community and facility‐level engagement in planning and budgeting for the government health sector—a district perspective from Kenya. Health Policy. 2011;99(3):234‐243. 20888061 10.1016/j.healthpol.2010.08.027 PMC4503225

[26] Gray A , Steele R . Programme budgeting in the health sector. Omega. 1979;7(5):451‐458.

